# Evaluation of the role of substrate and albumin on *Pseudomonas aeruginosa* biofilm morphology through FESEM and FTIR studies on polymeric biomaterials

**DOI:** 10.1007/s40204-017-0061-2

**Published:** 2017-02-02

**Authors:** S. Dutta Sinha, Susmita Chatterjee, P. K. Maiti, S. Tarafdar, S. P. Moulik

**Affiliations:** 1 Department of Physics, Jadavpur University, Kolkata, 700032 India; 2 Centre for Surface Science, Department of Chemistry, Jadavpur University, Kolkata, 700032 India; 3 Department of Microbiology, SSKM Hospital-Institute of Postgraduate Medical Education and Research, Kolkata, 700020 India

**Keywords:** Biofilms, Biomaterials, Adsorption, Bacteria, Proteins, Conditioning layer

## Abstract

**Abstract:**

Bacterial biofilms pose the greatest challenge to implant surgeries leading to device-related infections and implant failure. Our present study aims at monitoring the variation in the biofilm architecture of a clinically isolated strain and ATCC 27853 strain of *Pseudomonas aeruginosa* on two polymeric biomaterials, used in implants. The perspective of our study is to recognize the potential of these two biomaterials to create biofilm infections and develop the understanding regarding their limitations of use and handle patients with this deeper insight. The final goal, however, is an accurate interpretation of substrate-microbe interactions in the two biomaterials, which will provide us the knowledge of possible surface modifications to develop of an efficacious anti-biofilm therapy for deterring implant infections. The reference strain ATCC 27853 and a clinical isolate of *P. aeruginosa* collected from urinary catheters of patients suffering from urinary tract infections, have been used as microbes while clinical grades of polypropylene and high density polyethylene, have been used as ‘substrates’ for biofilm growth. The variation in the nature of the ‘substrate’ and ‘conditioning layer’ of BSA have been found to affect the biofilm architecture as well as the physiology of the biofilm-forming bacteria, accompanied by an alteration in the nature and volume of EPS (extracellular polysaccharide) matrices.

**Graphical Abstract:**

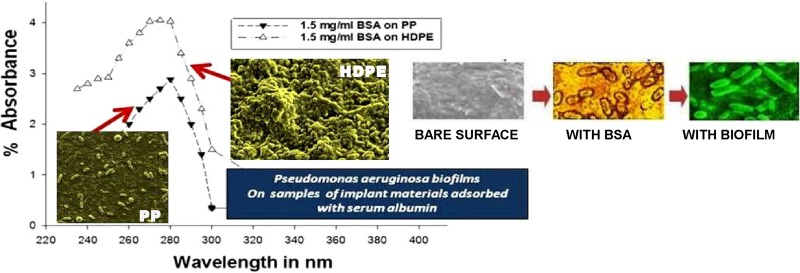

**Electronic supplementary material:**

The online version of this article (doi:10.1007/s40204-017-0061-2) contains supplementary material, which is available to authorized users.

## Introduction

**Fig. 1:**
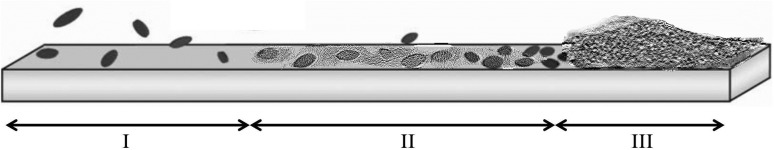
Stages of bacterial attachment to a biomaterial surface. *I* Adhesion of bacteria to biomaterial involves reversible cellular association with the surface. *II* Strong bacterial adhesion due to irreversible molecular bridging through cell surface adhesin compounds. *III* Biofilm formation

Indwelling medical devices such as catheters, heart valves, vascular bypass grafts, ocular lenses, artificial joints, cardiac stents, and central nervous shunts have become an integral and indispensable part of modern day clinical practice. These devices are responsible for reducing mortality and improving quality of life of patient. Unfortunately, submerged surfaces of these devices act as excellent seat for microbial biofilms (Donlan 2001; Costerton et al. 1999). The physicochemical forces that mediate bacterial adhesion can be divided into two time-dependent phases (Fig. 1). Phase I involves reversible bacterial attachment with the surface over the first 1–2 h post-implantation which is mediated through long-range (e.g., gravitational, van der Waals, and electrostatic interactions) and short-range (e.g., hydrogen bonding, dipole–dipole, ionic, and hydrophobic interactions) forces (Hori and Matsumoto 2010). Phase II begins approximately 2–3 h later, which is characterized by stronger adhesion between the bacteria and the implanted biomaterial resulting in irreversible molecular bridging (Li et al. 2012). Beyond Phase II, only certain bacterial strains are capable of forming a biofilm, when appropriately supplied with water and nutrients. Biofilms are consortium of surface adhered bacteria, which are embedded in a self-secreted matrix of extracellular polymeric substances (EPS) (Flemming et al. 2007; Flemming and Wingender 2010; Vu et al. 2009; Branda et al. 2005; Haggag 2010; Alpkvist et al. 2006; Wingender et al. 2012). Bacteria within biofilm are extremely resistant to actions of antimicrobials (Stewart and Costerton 2001; Mah and O’Toole 2001; Høiby et al. 2010) and can evade host immune reaction (Hornef et al. 2002; Foster 2005), making device-associated biofilm infections extremely difficult to treat (Weinstein and Darouiche 2001; von Eiff et al. 2005; Lynch and Robertson 2008).

The Gram negative bacterium *Pseudomonas aeruginosa*, an opportunistic pathogen (Whiteley et al. 2001; Campa et al. 2012; Oberhardt et al. 2008), is one of the most prevalent colonizer of medical devices in immuno-compromised patients (Kiehn and Armstrong 1990; Roy-Burman et al. 2001; Falkinham et al. 2015; Waldvogel and Bisno 2000). The objectives of the study were to evaluate the proneness of the surfaces of two widely used biomaterials toward biofilm formation, and identify the roles of *implant surface* and *conditioning layer* in modulating the biofilm architecture. The ultimate aim was, however, to gain an insight of the substrate-microbe interactions on the implant surfaces (Tuson and Weibel 2013; Dang and Lovell 2016; Salta et al. 2013; Hori and Matsumoto 2010), to enable the tailoring of surface properties, to control the emergence of biofilm formation on these surfaces, and potentially avoiding the occurrence of peri-implant infections. The field emission scanning electron microscopy (FE-SEM) has been used as a prime investigating tool and FTIR analysis was performed to compare the difference in the bacterial accumulation in the biofilms of the clinical and reference strains of *P*. *aeruginosa* on a single biomaterial surface.

Any solid surface when immersed in an aquatic environment gets adsorbed with the neighboring dissolved organic matter. Such a situation is similar to the placement of a biomaterial implant inside a biological fluid such as blood, plasma or urine which is followed by subsequent adsorption of proteins to the biomaterial surface (Huang et al. 2000; Rios et al. 2007). The adsorbed layer is defined as the ‘*conditioning layer*’ or ‘*molecular film*’ (Compere et al. 2001; Bakker et al. 2003; Bhosle et al. 2005; Garrett et al. 2008). The formation of the ‘*conditioning layer*’ modifies the surface characteristics of the *bare biomaterial surface*, due to change in its physical properties such as surface charge, wettability, hydrophobicity, and surface roughness (Amiji and Park 1993; Dewez et al. 1999; Hetrick and Schoenfisch 2006; Chu et al. 2002). All subsequent biological responses of the biomaterial surface, including antigenic response, attachment, and growth of cells and thrombosis, depend critically on the layer of adsorbed protein (Vogler 2012; Castner and Ratner 2002).

The effect of turbulence on nitrifying biofilms was studied in cylindrical PVC reactors (Kugaprasatham et al. 1992). Some of the approaches to limit bacterial colonization have focused on chemical degradation of stably adhered bacteria, including surface functionalization with microbicidal agents (Chang and Merritt 1992; Tiller et al. 2002). It was observed that the parameters defining substrate flux and biofilm structure (areal density, filament height, and cross-sectional area of filament) are inter-related parameters and are strongly affected by turbulence near the biofilm. In contrast to the widely held view that microorganisms respond rapidly to changes in environmental conditions, it has been observed (Freeman and Lock 1995) that the microbes in biofilms appear remarkably resilient to substantial changes in dissolved high-molecular-weight materials as it did not affect bacterial densities or the synthesis of phospholipids and DNA. The effect of different substrate loading rates and shear stresses on thickness, roughness, and density of mono-population *Pseudomonas aeruginosa* biofilms was observed by growing in an annular reactor (Peyton 1996). The effects of substrate and pH on biofilm nitrification were studied using a microelectrode technique and a micro-slicing technique (Zhang et al. 1999). It has been observed that numerous conditions, such as surface and interface properties, nutrient availability, the composition of the microbial community, and hydrodynamics, can affect biofilm structure (Stoodley et al. 1997). It has also been observed that under high shear stresses, such as on the surface of teeth during chewing, the biofilm (dental plaque) is typically stratified and compacted (Bowden et al. 1997; Wimpenny and Colasanti 1997). Efforts of controlling microbial biofilms on surfaces have been tried by surface impregnation with slow-releasing biocides such as gold or silver (Lee et al. 2005; Li et al. 2006; Saygun et al. 2006; Hetrick and Schoenfisch 2006) and antibiotics (Chang and Merritt 1992; Kohnen et al. 2003) or surface functionalization of specific antimicrobial peptides and polymers (Tiller et al. 2002; Etienne et al. 2005; Ignatova et al. 2006; Rudra et al. 2006). Since biofilm formation requires an initial stable attachment of a viable microbial population on a surface, a promising approach to limiting microbial colonization is prevention of bacterial adhesion to material substrata prior to colonization. Others have reported that poly(ethylene glycol)-conjugated polypeptides confer adhesion resistance and hypothesized that such results may be due to high degrees of substrata surface hydration (Boulmedais et al. 2004).

The effect of antimicrobials and alkali on biofilms was studied for different bacteria isolated from root canals with persistent infections (de Paz et al. 2010) with the help of confocal microscopy and a mini flow cell system, which was followed by image analysis. The biofilm system developed by them was sensitive to antimicrobials commonly used in endodontics but the effects were substratum-dependent, and most organisms displayed increased resistance to the antimicrobials on collagen-coated surfaces. The influence of glucose concentration and flow velocity on the distribution of effective diffusivity in biofilms was evaluated (Beyenal and Lewandowski 2000). It was observed that the Coulombic efficiency (CE) and power output from microbial fuel cells varied with different substrates, while the bacterial viability was similar for all the systems (Chae et al. 2009). It was noted that with increasing substrate COD/N ratios, the specific oxygen utilization rates of nitrifying bacteria in biofilm were found to decrease, indicating that nitrifying population became less dominant (Liu et al. 2010). It has been demonstrated (Zhu et al. 2004) that nitrate can serve both as a growth-controlling nutrient and as an electron acceptor in a biofilm for the respiration of VOCs with low Henry’s constants. Ammonium and nitrite are two substrates of anammox bacteria, but they are also inhibitors under high concentrations. The performance of two anaerobic ammonium-oxidizing (anammox) upflow biofilm (UBF) reactors was investigated (Tang et al. 2010). It was found that ammonium and nitrite are two substrates of anammox bacteria, but they are also inhibitors under high concentrations. Investigating the potential utility of d-amino acids in preventing device-related infections, it has been shown (Hochbaum et al. 2011) that surfaces impregnated with d-amino acids were effective in preventing biofilm growth. The initial biofilm formation on Ti implant surfaces with different micro-topography and hydrophilicity has been examined (Almaguer-Flores et al. 2012; Li et al. 2012), which reveal that initial biofilm formation and composition are affected by surface micro-topography and hydrophilicity.

It can be stated more generally that the development of a versatile and comprehensive approach to reduce stable bacterial adhesion to surfaces has been limited by incomplete understanding of the regulating physicochemical material properties and factors involved in the substrate–microbe interactions.

## Experimental

The present study focuses on the *modulation of biofilm architecture* of a clinical strain and a reference strain of *P*. *aeruginosa* (ATCC 27853), in relation to the interfacial properties of two polymeric biomaterials, which are widely used in implants and indwelling medical devices.

In our experiment, we have defined ‘*substrate’* as the surface of the biomaterial on which the biofilms are formed. This may be the *bare surface* of the biomaterial or the *biomaterial surface adsorbed with BSA*. The biomaterials, polypropylene (PP) used in a venous catheters and other indwelling medical devices and high density polyethylene (HDPE), frequently used in orthopedic implants have been used in our experiments as *substrates* for biofilm growth while the serum protein BSA is used as the *adsorbate*. Albumin (BSA in our case) has been chosen as the adsorbate, since concentration of serum albumin in the plasma is among the highest and ranges from 35 to 50 mg/mL, while it is only 0–5 pg/mL for interleukin 6. Also, according to Vroman effect (Leonard and Vroman 1992), albumin is one of the initial components to get adsorbed on an implant surface after its introduction at the implant site.

Though biofilms have been studied in detail from a variety of perspectives, the modulation in the patterns of biofilm formation in correspondence with properties of *substrate* or *conditioning layer* has never been duly characterized. Our research reveals that the architecture of biofilm formation by a single strain of bacteria varies in response to alteration of *substrate* and *conditioning layer*. The study consists of two parts: (1) adsorption of BSA on biomaterial surface to generate a *conditioning layer* (2) comparison of the morphologies of biofilms of a clinically isolated strain and a reference strain of *P*. *aeruginosa* on multiple biomaterials, widely used in implants.

### Biomaterials and production of the conditioning layer

Commercially available PP and HDPE having machined finish were obtained in square configuration (10 mm × 10 mm) from Plastic Abhiyanta Ltd, India. PP is used in a variety of catheters except urinary catheters, while HDPE is widely used in orthopedic implants. The water used in all our experiments was of HPLC grade (Lichrosolv) from Merck, India. Tris buffer was obtained from Sigma-Aldrich, USA while BSA was from MP Biomedical Ltd, USA. The polymer chips were initially cleaned in an ultrasonic cleaner, rinsed with water, autoclaved, blow dried, and preserved in a vacuum desiccator for adsorption experiments.

BSA was mixed in two different proportions with buffer solutions of pH 7.4 (concentration were 0.5 mg/ml and 1.5 mg/ml) and left for about a week with intermittent mixing to dissolve the BSA completely. BSA solution of a specific concentration was taken in six separate glass vials each containing a single chip for 9, 12, 15, 18, 21, and 24 h. These times are termed as exposure time ‘*τ*’ whose maximum (*τ* ^max^) and minimum (*τ* ^min^) were 24 and 9 h, respectively. After the stipulated time, the chips were removed from the protein solutions, rinsed with water, and finally blow-dried and preserved in a desiccator ready for growing biofilms. The chips obtained from adsorption experiments possess different degrees of BSA adsorbed on them, which is termed as the *conditioning layer*, and could not be sterilized further to prevent the denaturation of the adsorbed protein. The chips preserved in our experiments for the growth of biofilms were all adsorbed with [BSA] of 1.5 mg/ml to standardize the ‘conditioning layer’.

The BSA solutions obtained from adsorption experiments after the removal of each chip were preserved for at 4 °C for absorbance measurements. The UV absorbance was measured in a Shimadzu 2550 UV/VIS Spectrophotometer (Shimadzu, Japan) in matched 3.0 cm quartz cells.

### Bacterial strain and culture condition

One strong biofilm-forming clinical strain of *P*. *aeruginosa* isolated from the surface of a uro-catherter used for a prolonged period of time and one reference strain of *P*. *aeruginosa* ATCC 27853 were included in the study. After thawing, the frozen culture was adjusted to 0.5 McFarland standards. This suspension was diluted 1:100 and 1 ml was used to inoculate 100 ml of sterile LB broth (Luria Bertanii Agar (LBB) obtained from Himedia, India). The bacteria grown overnight in LBB at 37 °C were diluted in the same broth to an optical density of 0.5 at 600 nm and used as inoculums for biofilm study.

### Growing of biofilms

The treated and untreated chips were placed in the wells of 24 well tissue culture plate and 1 ml of bacterial culture (O.D 0.5 at 600 nm) were added to each well. Each 24 well plate contained a separate set of chips with a definite adsorption time and each system was closed and sealed without addition or removal of any component with the exception of broth. The sterile LBB was added carefully from time to time to avoid desiccation and incubated at 37 °C for 7 days with shaking at 180 rpm. Each set of experiment was performed on triplicate. The plates were sealed and placed on the shaker plate of the BOD incubator set at 180 rpm and rotated simultaneously for 7 days. Care was taken to ensure that each plate was in upright position during rotation, without tilting, which might affect the growth condition of the biofilms. After the entire 7-day growth period, the polymer chips were aseptically removed and washed thrice with phosphate buffered saline (PBS pH 7.2) to remove planktonic bacteria. Chips were then air dried and prepared for FESEM measurements.

The above protocol for biofilm growth was repeated for the reference strain of *P*. *aeruginosa* ATCC 27853 and the chips with biofilms were also prepared for FESEM measurements.

#### Sample preparation for FE-SEM

The chips with attached bacterial cells were covered with 2.5% glutaraldehyde and kept for 3 h in 4 °C after which they were washed thrice with the phosphate buffer solution. They were then passed once through the graded series of alcohol (25, 50 and 75%, twice through 100% ethanol) each for 10 min, finally transferred to the critical point drier and kept overnight to make them ready for biofilm analysis.

To compare the architecture of the biofilms produced by the clinically isolated strain of *P*. *aeruginosa* on different substrates after 7 days, FESEM measurements were conducted at 2.0–10 kV in a field emission scanning electron microscope (FESEM: Inspect F50, FEI Europe BV, and The Netherlands; FP 2031/12, SE Detector R580). For this purpose, the dried polymer chips with and without biofilms were sputter-coated with a 3-nm-thick conductive layer of gold. We have used Image J for analysis of the SEM images.

## Results

### Adsorption experiments

The BSA solution preserved in each vial after removal of the polymer chips obtained from “Biomaterials and production of the conditioning layer” section was subjected to absorbance measurements in a UV–visible spectrophotometer. For all solutions, peaks of absorbance at 270–280 nm with varying intensities were observed. Considering a particular polymer, say PP (Figure SI 1, Supplementary Information) for a fixed exposure time, absorbance was found to be higher for higher initial [BSA].

Hence, higher absorbance implied higher concentration of residual BSA solutions, which has been verified by our experimental results. Higher exposure time had a decreasing effect on the absorbance at each initial concentration. Thus, at each [BSA], higher exposure time produced more adsorption of the protein on to the surface of the polymer chips proving that the adsorption of BSA was proportional to [BSA] in solution for the same exposure time irrespective of the mechanism of adsorption involved.

Comparison between the absorbance peaks of PP and HDPE (Figure SI 2, Supplementary Information) having the same adsorption time (both 18 h), and equal [BSA] showed higher peaks of the latter compared to the former. Thus, we can conclude that under similar conditions of pressure, temperature, and pH, the adsorption of BSA was higher on the surface of PP than on HDPE.

### Growth of biofilms

#### Effect of substrate

**Fig. 2:**
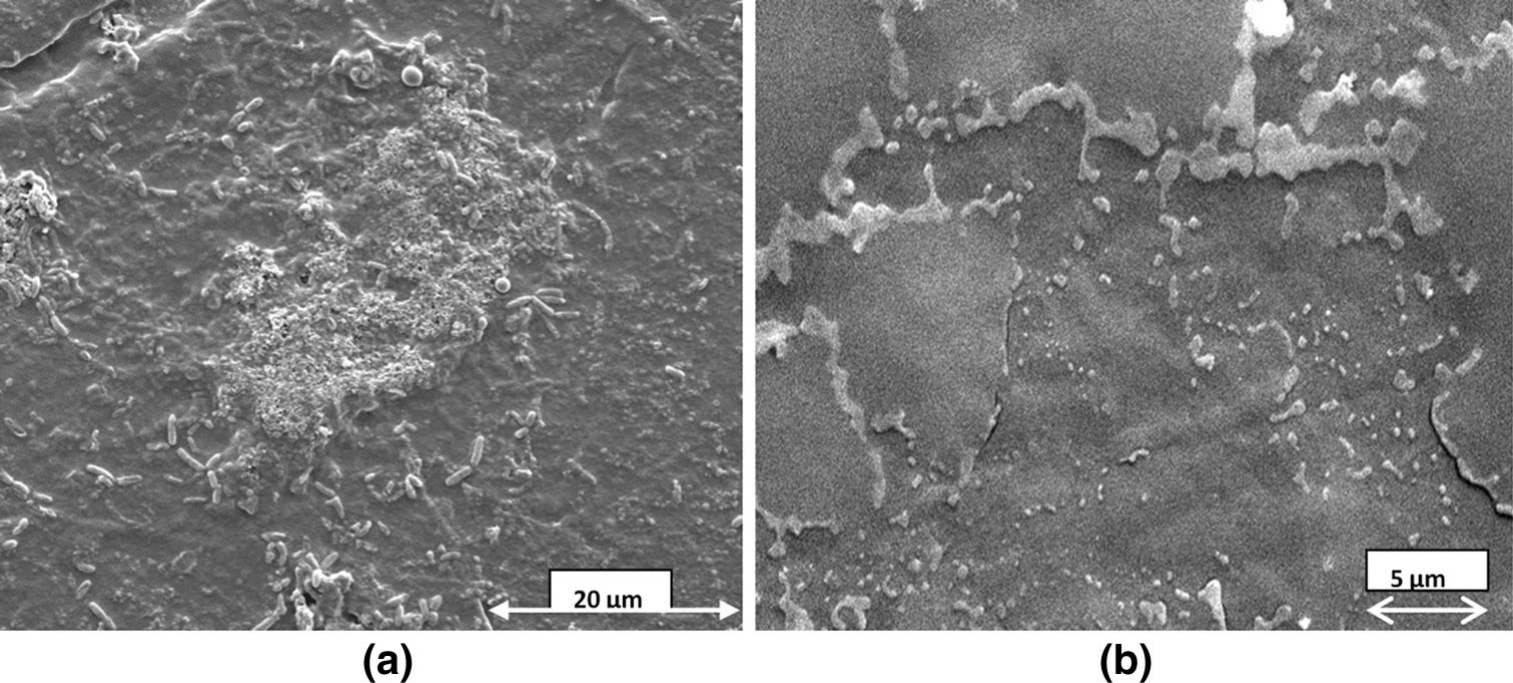
FESEM images on PP surface adsorbed with BSA for 24 h by *Pseudomonas aeruginosa* **a** clinical strain and **b** reference strain ATCC 27853

The FESEM images of the biofilms formed by the clinical and ATCC strains of *P*. *aeruginosa* on a pristine PP surface adsorbed with BSA for 9 h are shown in Fig. 2. The spread-out nature is common to both biofilms in Fig. 2a, b, citing a similarity in response of the two strains of bacteria to the same substrate (i.e., PP surface with conditioning layer of BSA) regarding biofilm adhesion. Though both biofilms are 7 days old, in the former image (Fig. 2a) bacteria are found dispersed from the mature biofilm (shown with black arrow), but in the latter (Fig. 2b) biofilm architecture is such that all bacteria remain concealed. Pores and channels are characteristic of the biofilm in Fig. 2a, while biofilm of Fig. 2b is significantly dominated by flat surfaces (resembling salt flats), with the presence of NaCl as indicated by EDAX studies (Fig SI 3, Supplementary Information).

**Fig. 3:**
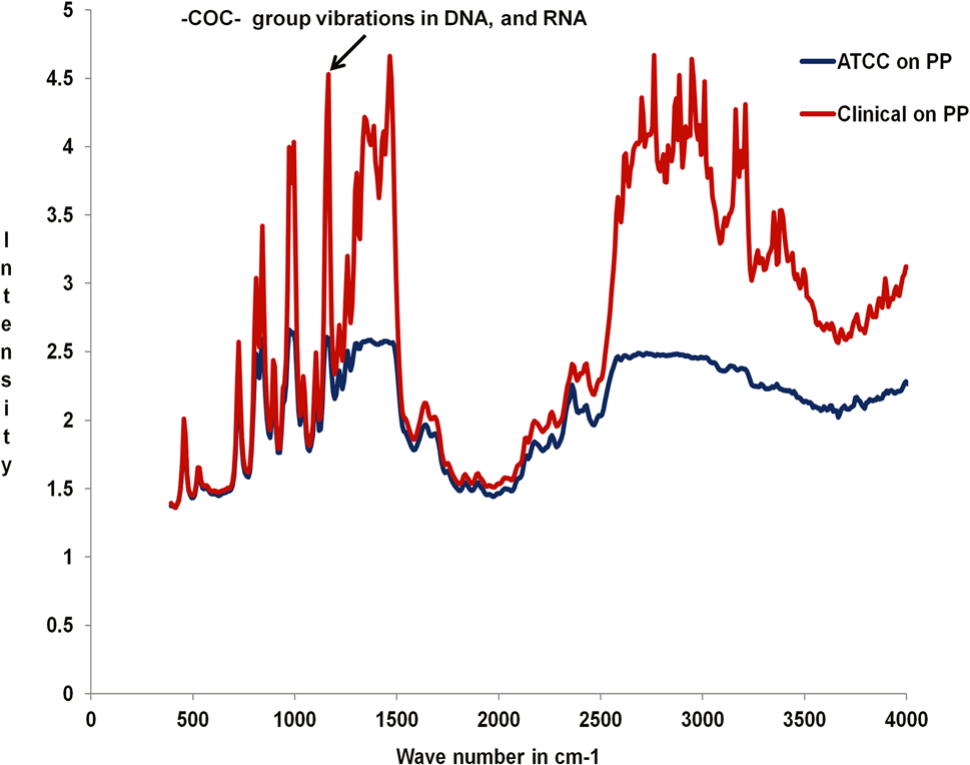
FT-IR studies of biofilms produced by clinical and ATCC strains of *P*. *aeruginosa* on PP surface adsorbed with BSA for 24 h

The presence of the bacteria concealed within the biofilm of almost flat topography was revealed through FT-IR data (Fig. 3) showing presence of nucleic acids (Jiao et al. 2010). It is evident from the comparison of FT-IR data of EPS matrices of the respective strains of bacteria that very little/no differences do exist between them with regard to their chemical composition, proportion of each chemical constituent but significantly in the amount of bacteria embedded within the respective biofilms. We have not performed a detailed analysis of FTIR data here, but a glimpse of the similarity and differences is only provided to verify the effect of different bacteria on a single substrate. However, the FE-SEM images in Fig. 2a, b reveal almost identical spreading and adhesion of the EPS matrices of both strains on the BSA-adsorbed PP surface.

**Fig. 4:**
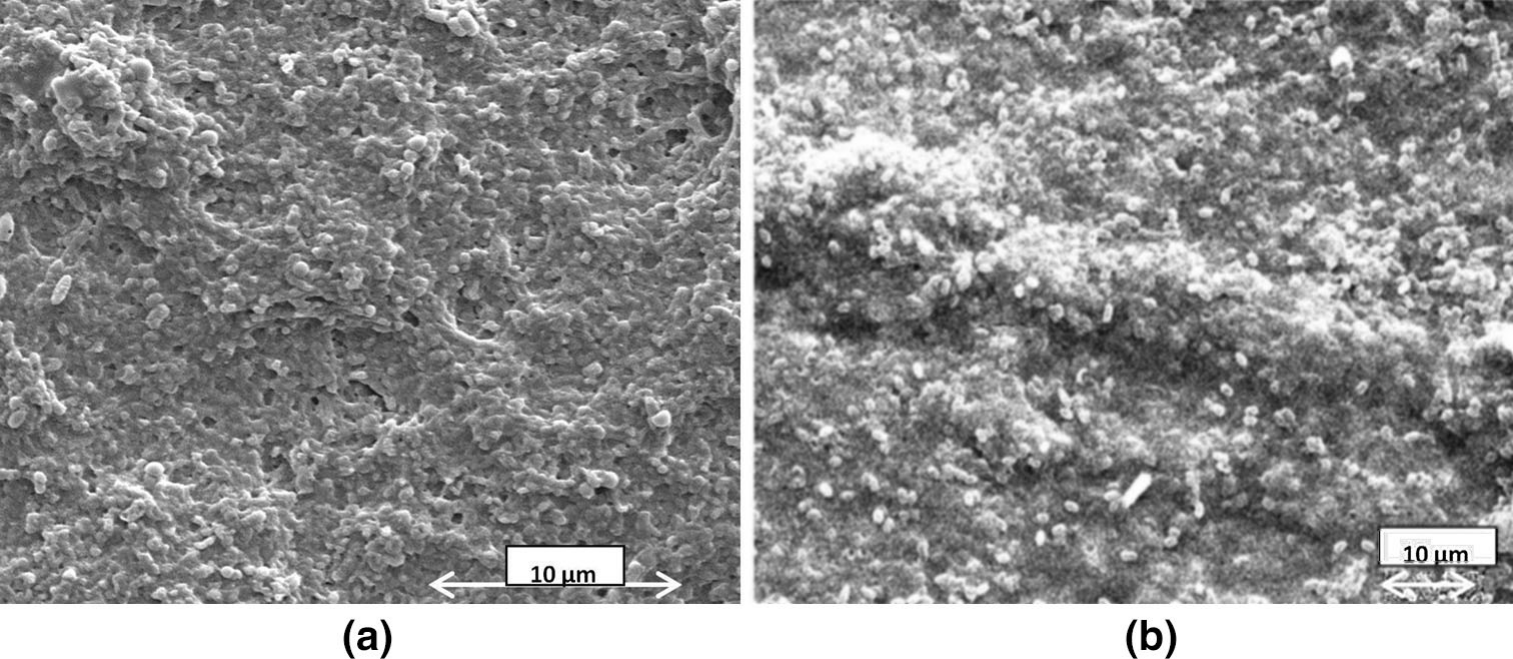
FESEM images of 7-day old biofilms of *P*. *aeruginosa* with **a** clinical strain, **b** ATCC strain

A similar comparison of FE-SEM images of the biofilms formed by the clinical and ATCC strains of *P*. *aeruginosa*, on a pristine HDPE surface adsorbed with BSA for 24 h is shown in Fig. 4a, b. The biofilms for both strains in this case have a undulating surface and dispersed biofilm bacteria are clearly visible in Fig. 4a, b. Both biofilms show abundant pores and channels typical of a true biofilm unlike that in Fig. 2b, which appears to be a stack of microbial cells and no trace of pores and channels on its surface. Another common feature revealed by the above images is the altered physiology of the biofilm bacteria on the HDPE surface. A comparison of biofilms in Figs. 2a, b and 4a, b demonstrate that bacterial cells of both the strains of *P*. *aeruginosa* react physiologically to the variations in the properties of the respective *substrates*, by altering their physical appearance and simultaneously regulating the nature and quantity of the secreted extracellular polymers. *P*. *aeruginosa*, which is normally a rod-shaped bacteria, as also observed in the biofilm on PP surface in Fig. 2a, transforms to a bean-shaped morphology, on HDPE surface adsorbed with BSA for 24 h as observed in Fig. 4a, b. Hence, both strains of bacteria possess an identical property of interacting in response to the substrates and in a similar way.

Hence, it can be concluded that comparison of the morphologies of the biofilms produced by the clinical and reference strains of *P*. *aeruginosa* on a PP surface adsorbed with BSA reveals a similarity in spreading and adhesion with the substrate, but they have visible differences in their respective architecture. On the other hand, the same strains of bacteria on HDPE surface adsorbed with BSA produce biofilms which have similar adhesive and morphological properties. The FTIR data in Fig. 3 reveal that though the chemical constituents of the EPS matrices for both the strains on the PP surface are roughly similar, the amount of bacteria embedded within the biofilms grossly differs.

#### Effect of adsorption time

**Fig. 5:**
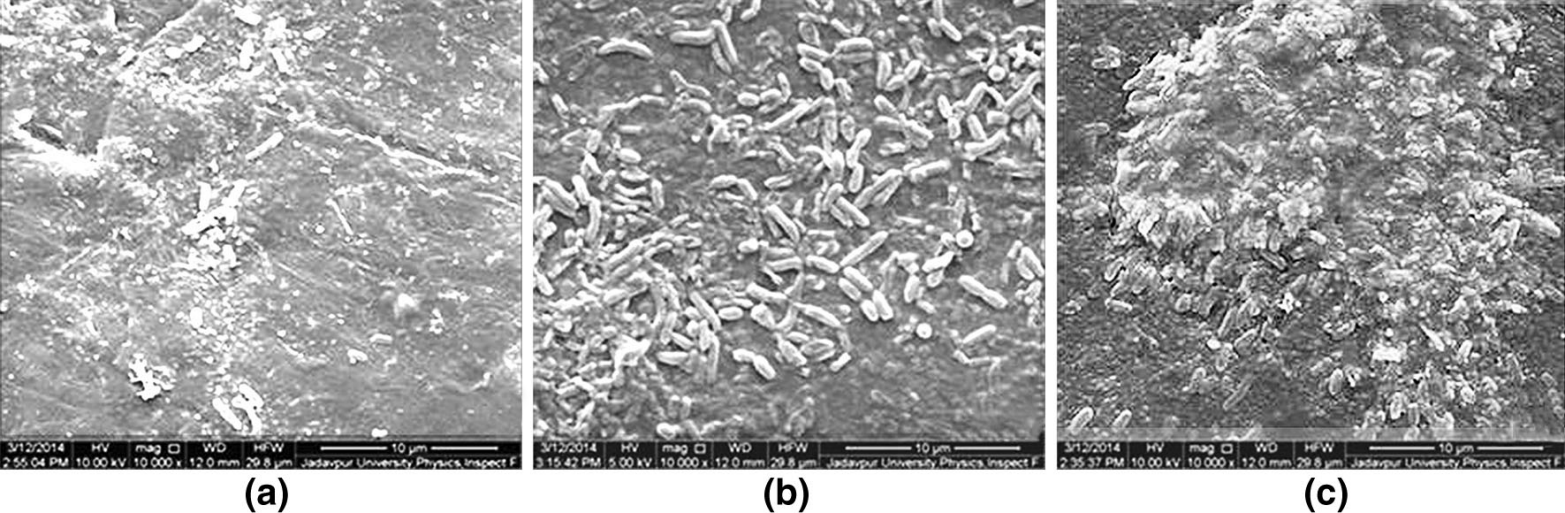
FESEM images of 7-day old biofilms of *P*. *aeruginosa* on PP surface. **a** Bare surface, **b** with BSA adsorbed for 9 h, **c** with BSA adsorbed for 24 h

The FESEM images of the biofilms formed by the clinical strain of *P*. *aeruginosa*, on a pristine PP surface and on those adsorbed with BSA for 9 and 24 h duration, are shown in Fig. 5a–c.

**Fig. 6:**
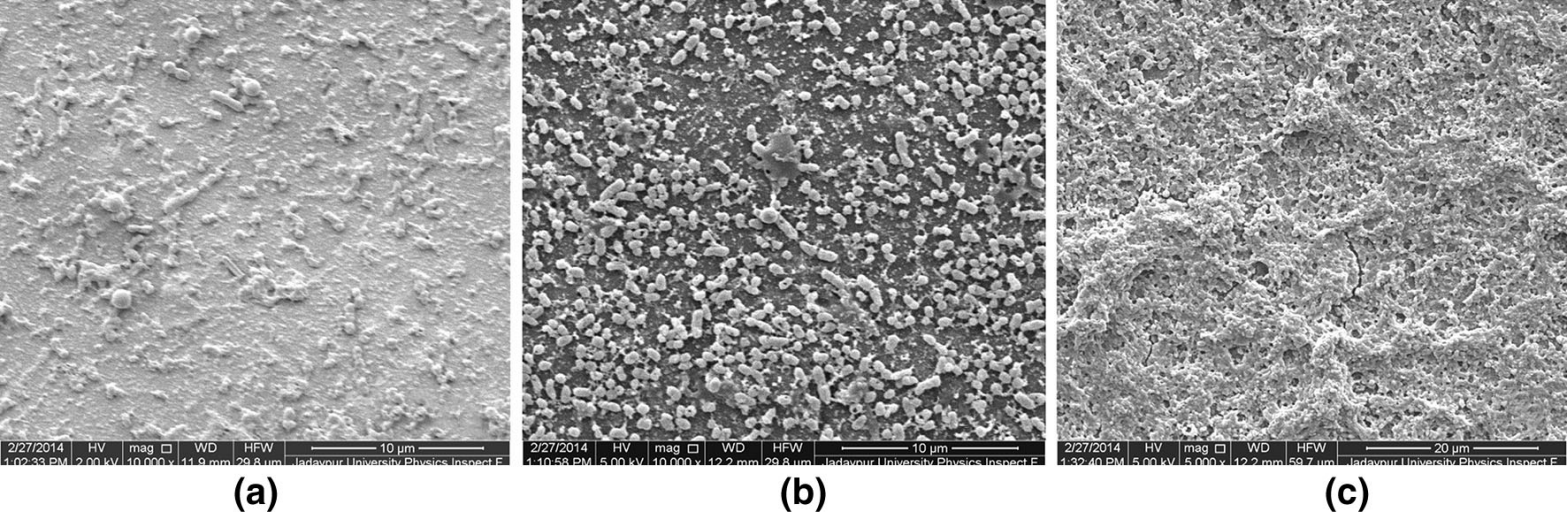
FESEM images of 7-day old biofilms of *P*. *aeruginosa* on HDPE surface. **a** On the bare surface, **b** with BSA adsorbed for 9 h, **c** with BSA adsorbed for 24 h

FE-SEM images of the biofilms of the same strains of bacteria and of similar age on pristine HDPE surface, and on those adsorbed with BSA for 9 and 24 h, are shown in Fig. 6a–c. Comparison of the biofilms in the respective situations in Figs. 5 and 6 reveals differences in their architecture in response to different substrates. However, repetition of biofilm growth on similar situations yields identical results.

The two-dimensional surface profiles (performed with Image J) of bare PP and HDPE surfaces (Fig SI 4, Supplementary Information) reveal differences of the order of 5–7% between the two, proving that they possess roughly similar topography. A comparison between the two-dimensional surface profiles of the 7-day old biofilms formed by the clinical strain of *P*. *aeruginosa*, on HDPE and PP adsorbed with BSA for 24 h (Fig SI 5, Supplementary Information), indicate a roughly uniform spread of the biofilm on the HDPE surface in contrast to a ‘focal adhesion’ in case of PP surface. The biofilm bacteria, however, followed the same trend on both the substrates, i.e., (biofilm formed on pristine surface) < (biofilm formed on BSA covered surface).

**Fig. 7:**
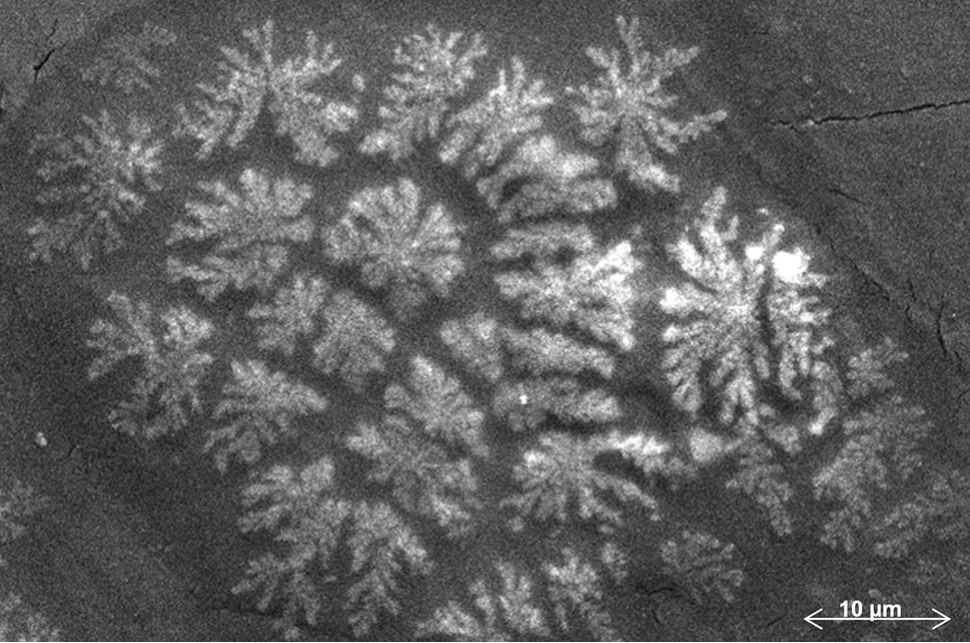
Patches of dendritic patterns of bacterial growth found on PP surface adsorbed with BSA apart from biofilm covered areas

Apart from the prevalent biofilm architecture of *P*. *aeruginosa* ATCC 27853 on the PP surface as depicted in Fig. 2, parts of the substrate are found to be covered with patches of dendritic bacterial growth as shown in Fig. 7. However, the normal biofilm architecture on PP is never accompanied or interspersed by the dendritic patterns but each patch happens to occur at distinctly different areas of the substrate. The biofilm of the clinical strain, however, does not have any fragment of dendritic growth throughout the PP substrate.

This phenomenon is also totally absent in ATCC/clinical strain biofilms on HDPE, where the biofilm morphology remains almost uniform (Fig. 4b) throughout the substrate.

## Discussion

The phenomena observed during the adsorption experiments on PP and HDPE surfaces (Fig SI 1 and Fig SI 2, Supplementary Information) can be explained in terms of hydrophobicities of the respective biomaterials and the strength of the non-covalent interactions between the biomaterial surfaces and the adsorbed protein layer. PP is more hydrophobic than HDPE, and the soft protein BSA prefers the PP surface more than HDPE which may also be supported from the perspective of critical surface tension (Baier 2006). PP has higher water contact angle compared to HDPE (Table 1), but its critical surface tension is lower than HDPE causing greater affinity for the adsorption of BSA on it. Also the water contact angle on PP adsorbed with BSA for 9 and 24 h are less than that on HDPE for similar adsorption times. In general, hydrophobic interaction significantly contributes to protein adsorption on a surface. By modifying the hydrophobic polystyrene latex surface to hydrophilic, the amount of BSA adsorbed was found to decrease (Nakanishi et al. 2001; Imamura et al. 2008; Alava et al. 2013).

**Table 1:** Variation of water contact angles with adsorption of BSA

Polymers (clinical grade)	Contact angles		
	Bare	BSA 9 h	BSA 24 h
PP	96.6865	93.746	87.22565
HDPE	95.2918	94.39885	92.97118

It is clearly evident from our experiments that the adsorbed protein, the strain of biofilm forming bacteria and exposure time *τ*, though same in the Figs. 2a and 4a and also in Figs. 2b and 4b, the cumulative properties of the substrate (surface with adsorbed BSA) grossly affect not only the morphology of the biofilm but also the anatomical and physiological features of the biofilm-forming bacteria in each case. The biofilm images in Figs. 5 and 6 separately reveal the effect of the exposure time *τ*. A comparison of the biofilms in the respective situations in Figs. 5 and 6 reveals a complete variation of biofilm architecture on the two substrates. The reason for variation in biofilm architecture on two different substrates under similar circumstances (such as strain of bacteria, conditioning layer, temperature, pH, and amount of shear stress) may be related not only to the differences in water contact angles on BSA-adsorbed PP and HDPE (refer Table 1), but also to the distinction in the nature of the interfacial properties of the substrates and substrate-microbe interactions. In biological systems, hydrophobic interactions are specifically the strongest long-range non-covalent interactions (van Oss 1997). Such interactions can be defined as the primary attraction between apolar or slightly polar molecules, particles or cells, when immersed in water and the hydrogen bonding energy of cohesion between the surrounding water molecules forms the sole driving force for this interaction. The residual hydration of HDPE (i.e., the Lewis AB forces) is responsible for the orientation of water molecules adsorbed on its surface. Hence, water molecules oriented on its surface will repel the water molecules having same orientation on the surface of adjacent particles (van Oss and Good 1991; Carvalho, et al. 2013) preventing complete adhesion. On the other hand, the bacterial cell surfaces, which are less polar (i.e., capacity for orienting the most closely adsorbed water molecules is less pronounced), approach each other under the influence of their net Lifshitz–van der Waals (LW) attraction forming a close network and robust biofilm. On the PP surface, however, the biofilm–substrate adhesion is extremely pronounced, due to its greater hydrophilicity after BSA adsorption (Table 1) than HDPE and squeezing out of water molecules from its surface during protein adsorption. Hence, biofilm–substrate adhesion in PP exceeds intercellular attraction as is evident from Fig. 4c. The above analysis explains the difference in the biofilm architecture observed on HDPE and PP surfaces.

Our experimental results lead us to conclude that there is a significant contribution of both the substrate and conditioning layer on the biofilm architecture, which may affect the degree of attachment of the bacteria to the respective biomaterial surfaces. The above two factors controlling biofilm architecture are also found to modulate the bacterial physiology, which might play a significant role in altering their pathogenicities and toxicities, apart from their EPS producing capacities. Such an attribute is impossible in a planktonic bacterium forming a colony on different *substrates*. It is thus imminent that the macromolecular components of the bacterial cell surfaces, e.g., lipopolysaccharide and protein, and exopolymers secreted during biofilm formation possibly vary in quantity and chemical composition with the variation in the interfacial properties of the substrate and the microbial receptors interact with the substrate accordingly varying their physical form. This clearly indicates a combined role of both the adsorbed protein and the biomaterial surface in altering the physiology of the biofilm-forming bacteria.

The appearance of patches of dendritic growth in the *P*. *aeruginosa* ATCC strain biofilm on the BSA-covered PP substrate is an interesting phenomenon and may be analyzed from the point of view of rhamnolipid production and display of group behavior. *Pseudomonas aeruginosa* is capable of twitching, swimming, and swarming motility (Caiazza et al. 2005). Among these, swarming motility is a group behavior that requires rhamnolipid biosynthesis. The result of swarming in *P*. *aeruginosa* is the complex patterns of cells organized as radiating tendrils, the spaces between which may be analogous to biofilm channels in that they remain uncolonized. It has been noticed in swarming experiments that tendrils of a given swarm rarely intersect, and furthermore, tendrils from different swarms change course as they approach each other (as in Fig. 1A in Caiazza et al. 2005). Also, when tendrils of opposing swarms approach, they change direction and swarm parallel to each other instead of crossing paths. The dendritic growth pattern in Fig. 7 reveals a similar characteristic as described above, indicating the probability of production of rhamnolipids in the ATCC biofilm on PP substrate. Such occurrence has not been observed in either of the other bacteria–substrate interactions presented here. The reason for the rhamnolipid production on a particular substrate or for a particular bacteria–substrate pair is, however, not known at present and may be revealed through future research. However, it might be predicted that the presence of rhamnolipids in the EPS of a biofilm is able to change the biofilm architecture due to their tensioactive properties (Beal and Betts 2000).

## Conclusions

In our experiments, the degree of adsorbed BSA is found to play a significant role in the growth and adhesion of the *P*. *aeruginosa* biofilm on both PP and HDPE. However, previous observations revealed that albumin is normally associated with inhibition of biofilm formation (An and Friedman 1998). Coating of polystyrene plates with Human serum albumin (HSA) was found to significantly reduce bacterial adhesion and biofilm formation in *S*. *pneumonia* (del Prado et al. 2010). But a few experimental evidence exist which reveal that anti-adherence effect is species-dependent, as albumin coating of titanium surfaces decreased the adhesion of *S*. *mutans*, but adhesion of *P*. *gingivalis* and *F*. *nucleatum* remained unaffected (Badihi Hauslich et al. 2013). This latter trend may be in agreement with our observations with both clinical and reference strains of *P*. *aeruginosa*.

It is hence evident that for *P*. *aeruginosa*, the nature of the substrate and conditioning layer regulates the possibility of formation, architecture, and adhesion of biofilms to medical implants, giving rise to acute biomaterial-associated infections (BAIs). A dense biofilm shown in Figs. 5c and 6c brings forth the vulnerability of infection in orthopedic implants and venous catheters. Moreover, greater volume of EPS of the biofilm ensures greater chance of thrombus formation within the blood vessels, after the maturation and detachment of the biofilm from the implant surface. The biofilms formed by the clinically isolated strain also prove beyond doubt that a strain of bacteria forming biofilms on urinary catheters (i.e., on silicon rubber) is equally capable of forming biofilms on any other *substrate*. Hence, while it is well known that *P*. *aeruginosa* is responsible for causing cystic fibrosis (CF), urinary infections, otitis media, and burn infections, it is quite obvious from our experiments, that the same pathogen might infect orthopedic implants or central venous catheters if it gets access to the same.

We can, hence, conclude from our studies that the bacterial receptors are able to identify the differences in the properties of the *substrates*, and modulate their genetic expressions accordingly. The mechanism of interaction may be different for different bacteria–biomaterial–protein combinations but the understanding obtained by various available techniques (Fattinger 2014; Vo-Dinh 2014) would further increase the existing knowledge of prevention of biofilm formation on implants. The alteration in the physiology of the biofilm-forming bacteria on encountering different substrates might be linked to a change in its genetic expression and not an emergence of a new genome. Further, gene sequencing studies in this regard will reveal the truth of this hypothesis. Employing further strategic studies and intensive research in this area in future might enable the creation of superb biomaterial surfaces that would be able to modulate the genetic expression of bacteria to a non-biofilm mode, irrespective of adsorbed conditioning layer.

## Electronic supplementary material

Below is the link to the electronic supplementary material.

Supplementary material 1 (DOC 3386 kb)
