# Supplementary material for: Evaluation of the role of substrate and albumin on Pseudomonas aeruginosa biofilm morphology through FESEM and FTIR studies on polymeric biomaterials
Source: Prog Biomater. 2017 Feb 2;6:27–38. doi: 10.1007/s40204-017-0061-2 (PMC5433955; doi:10.1007/s40204-017-0061-2)
Supplement: Supplementary file 1 — Supplementary material 1 (DOC 3386 kb) [file 40204_2017_61_MOESM1_ESM.doc]

**An evaluation of the role of substrate and albumin *on Pseudomonas aeruginosa* biofilm morphology through FE-SEM and FTIR studies on polymeric biomaterials**

Authors:

*S Dutta Sinha*^1^, Susmita Chatterjee^3^, P.K.Maiti^3^, S.Tarafdar^1^, S.P.Moulik^2^*

^1^ Department of Physics, Jadavpur University, Kolkata-700032, India

2 Centre for Surface Science, Department of Chemistry, Jadavpur University, Kolkata-700032, India

^3^Department of Microbiology, SSKM Hospital-Institute of Postgraduate Medical Education and Research, Kolkata-700020, India

**SUPPLEMENTARY INFORMATION**

**
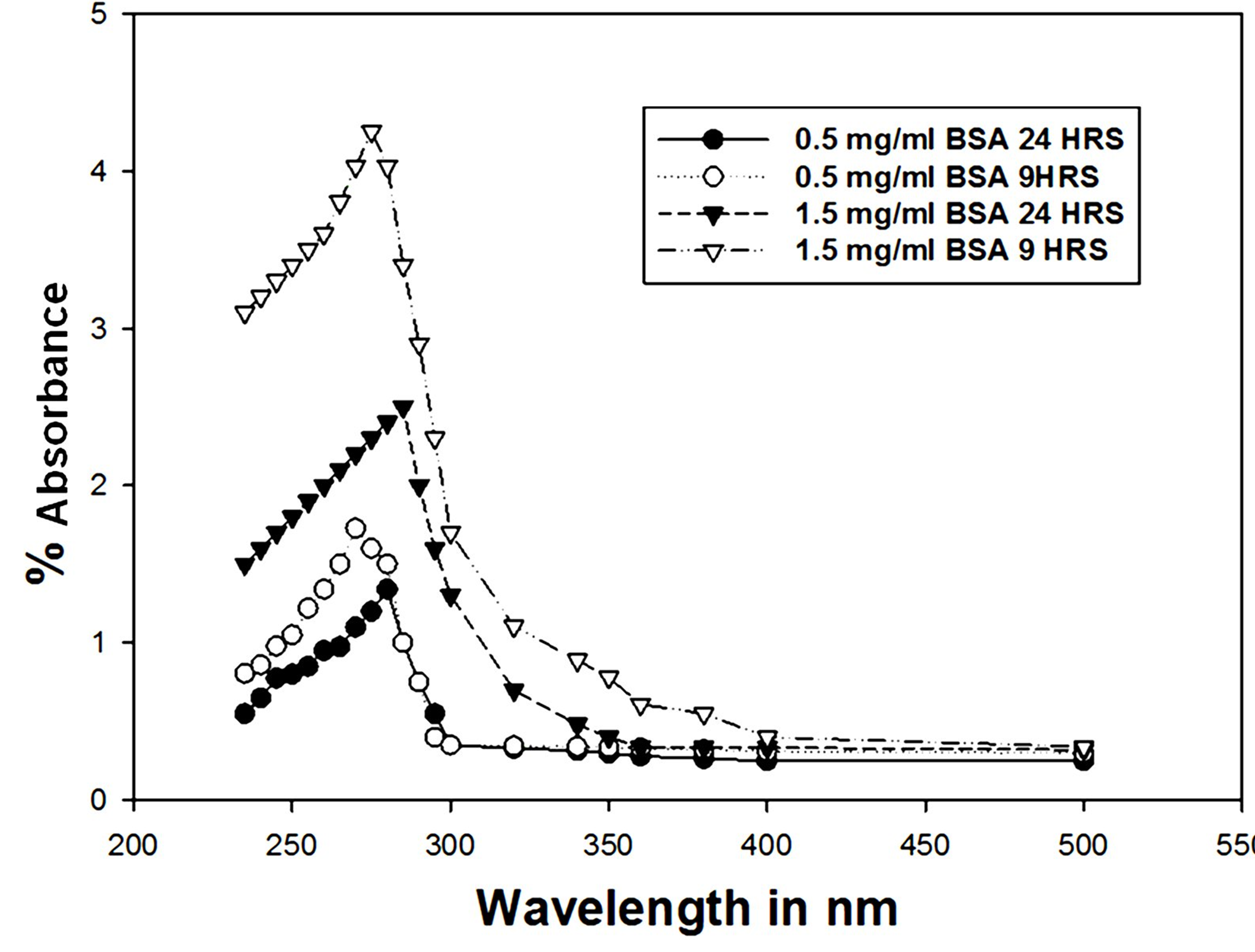
**

**Fig SI 1: Absorption-wavelength profiles of BSA of varied concentrations on PP after 9 and 24 hrs.**


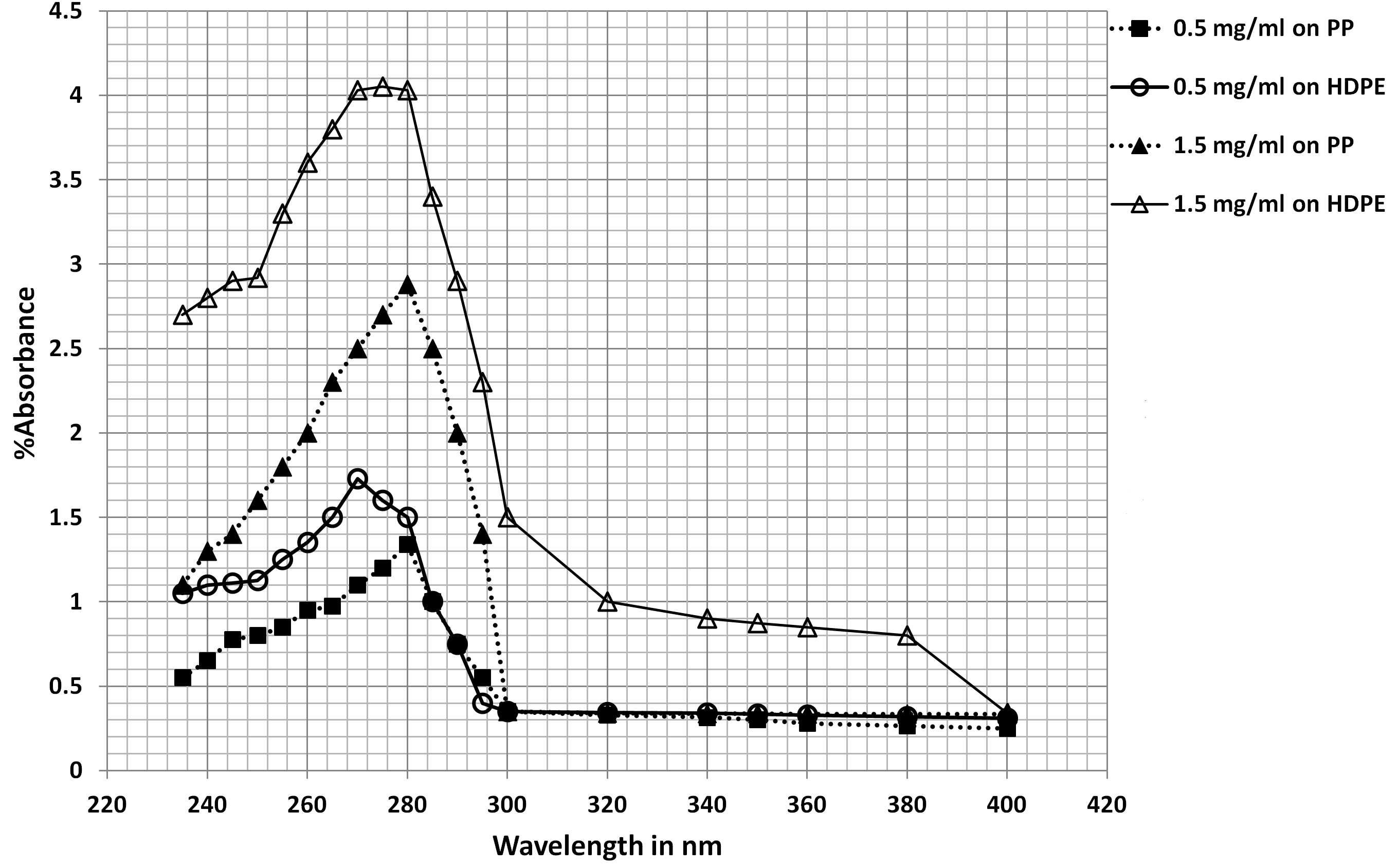


**Fig SI 2. Comparison of % absorbance of BSA on PP and HDPE.**


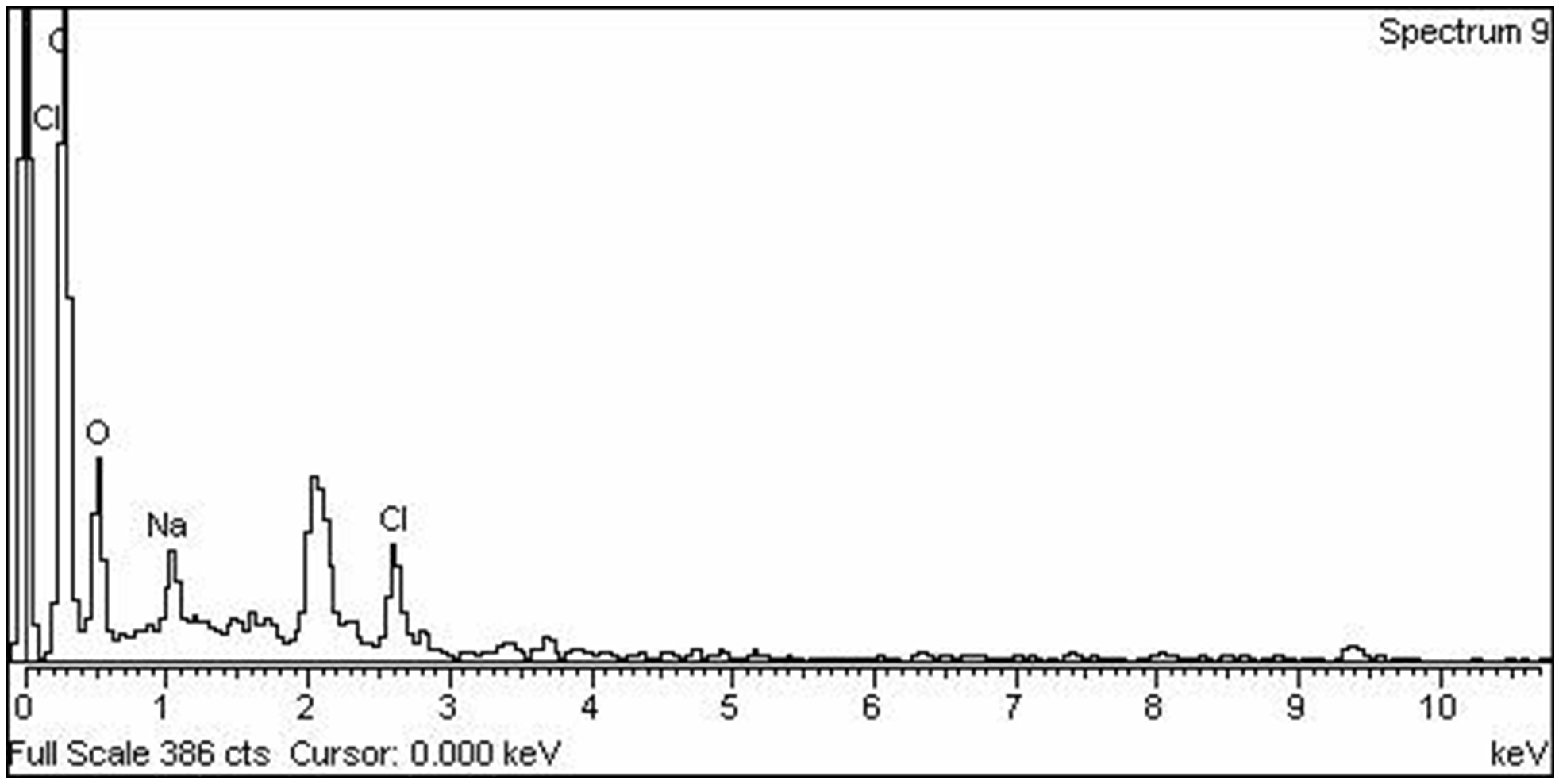


**Fig SI 3. EDAX results of P aeruginosa ATCC 27853 on BSA coated PP surface.**


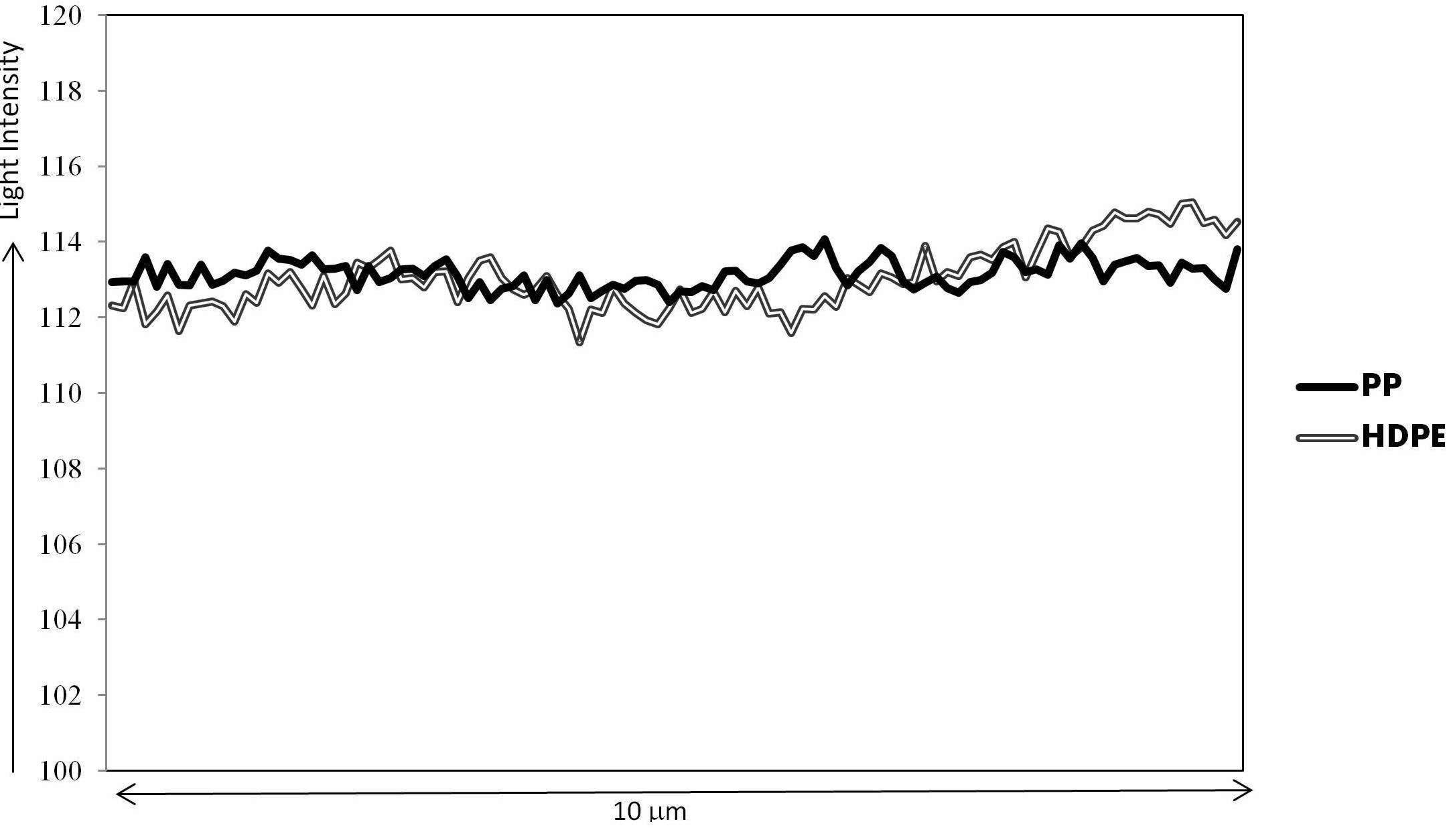


**Fig SI 4. Comparision of two dimensional surface profiles of bare surfaces of PP and HDPE**

**
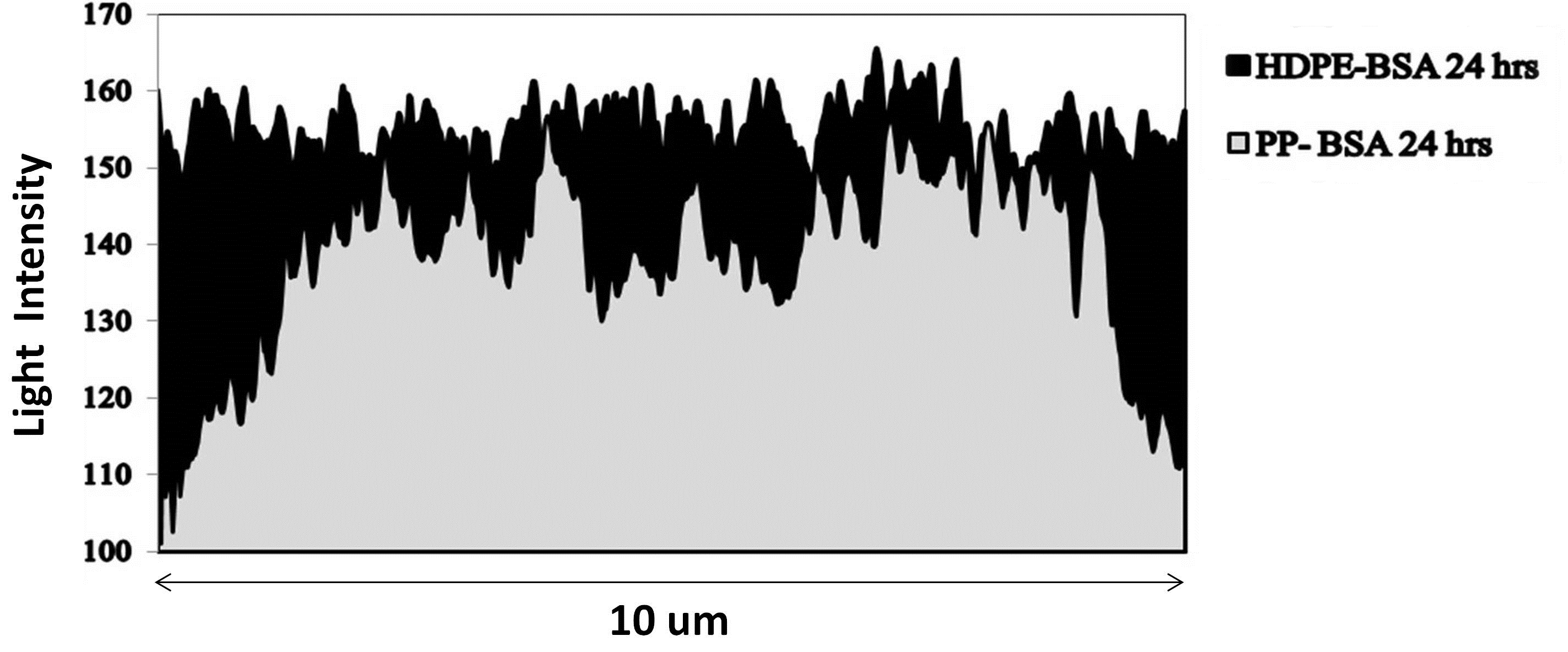
**

**FigSI 5. Comparision of two dimensional surface profiles of biofilms on PP and HDPE surfaces adsorbed with BSA for 24 hours**

**
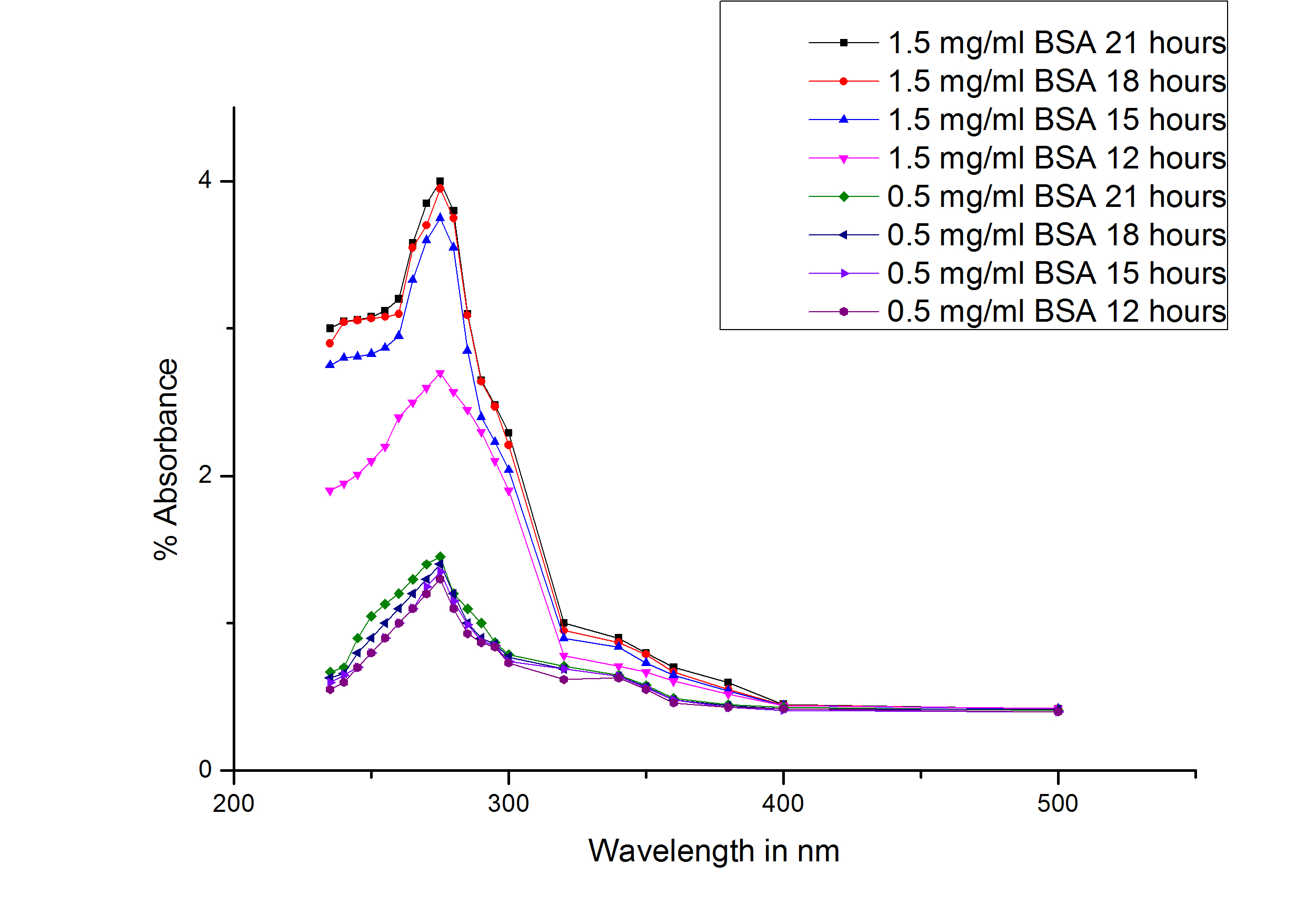
**

**Fig SI 6. Absorption-wavelength profiles of BSA of varied concentrations on PP after 12 hours, 15 hours, 18 hours and 21 hrs.**
